# The Association Between Active Membership in Voluntary Organizations and Homonegativity

**DOI:** 10.7759/cureus.60045

**Published:** 2024-05-10

**Authors:** Amirhossein Khanehpaza, Krit Pongpirul, Atthanee Jeeyapant

**Affiliations:** 1 Health Policy, School of Global Health, Chulalongkorn University Faculty of Medicine, Bangkok, THA; 2 Public Health, Department of International Health, Johns Hopkins Bloomberg School of Public Health, Baltimore, USA; 3 Health Policy, Clinical Research Center, Bumrungrad International Hospital, Bangkok, THA; 4 Health Policy, Center of Excellence in Preventive and Integrative Medicine, Chulalongkorn University Faculty of Medicine, Bangkok, THA; 5 Medicine, Mahidol Oxford Tropical Medicine Research Unit, Mahidol University Faculty of Tropical Medicine, Bangkok, THA

**Keywords:** voluntary activity, life satisfaction, volunteerism, homosexuality, homonegativity

## Abstract

Objectives: Homonegativity adversely affects the health and well-being of homosexuals in society, making it vital to identify factors associated with it. This study investigates whether active membership in voluntary organizations correlates with homonegativity, examining how this varies by gender and age.

Methods: Using the World Values Survey data (2017-2022) from 87,777 participants in 63 countries, we performed binary logistic regression to assess relationships between homonegativity and factors including socioeconomic status, demographics, and voluntary activity participation.

Results: Our findings suggest that active membership in certain voluntary organizations correlates with homonegativity levels among both men and women across various age groups. Specifically, active participation in sports or recreational organizations, professional associations, art, music, or educational organizations, and humanitarian or charitable organizations was found to be negatively correlated with homonegativity in specific gender-age groups, albeit with varying degrees of association strength.

Conclusion: This study highlights the complex relationship between engagement in voluntary organizations and attitudes toward homosexuality, with significant differences observed across gender and age. While identifying a correlation rather than causation, this study suggests the importance of societal and community participation in fostering more tolerant views toward homosexuals. Furthermore, our analysis indicates that demographic and socioeconomic variables, the political freedom of the respondent's country, and the respondent's life satisfaction are also linked to homonegativity.

## Introduction

The term "homonegativity" refers to negative reactions or attitudes towards homosexuality [1]. Assessing the levels and determinants of homonegativity within societies is crucial, as numerous studies have indicated its severe impact on the health and well-being of homosexuals [2-4]. For instance, homosexuals who face homonegativity often grapple with feelings of shame and self-loathing, leading to isolation [2], depression, anxiety, poor mental health [3], as well as alcohol abuse and even suicide [4]. Given the profound consequences of homonegativity on the well-being of homosexuals, it is imperative to understand its determinants. Past research has highlighted several factors influencing homonegativity, including religion, ethnicity, gender, education level, economic development, and more [5-9]. In this study, our objective is to determine whether active membership in voluntary organizations is linked to homonegativity.

Literature review

Volunteerism is defined as engagement in activities without expecting financial compensation [10]. The existing research exploring the relationship between volunteerism and homonegativity is relatively limited. A significant study highlighted the connection between homophobia and voluntary participation in extracurricular activities among high school students [11]. The results suggested that male students who engaged in mainstream sports (e.g., football, basketball) were more likely to exhibit negative attitudes towards homosexuality compared to their counterparts who didn't participate in such sports. Conversely, female students who participated in non-athletic extracurricular activities, like debate and science clubs, were less likely to harbor homonegative sentiments than those who didn't engage in these activities [11]. These findings underscore the influence of both gender and the nature of voluntary activity in shaping the correlation between volunteering and attitudes towards homosexuality.

Research questions

Can participation in voluntary activities predict homonegativity? Does the relationship between voluntary activities and homonegativity vary based on the type of activity? Does gender influence the relationship between voluntary activities and homonegativity? Is the relationship between voluntary activities and homonegativity consistent across all age groups?

This article was previously posted to the medRxiv preprint server on March 09, 2024.

## Materials and methods

The study received IRB approval from Chulalongkorn University, Faculty of Medicine (IRB Number: 0683/66). For this study, we utilized secondary data. The World Values Survey (WVS) stands as the most expansive non-commercial academic social survey program [12]. Since its inception in 1981, it has conducted surveys across over 120 countries, boasting over 60,000 citations in Google Scholar alone [12]. WVS provides freely downloadable data sets for various countries on their website. We secured permission to use their data through email correspondence. Our analysis centers on the latest World Values Survey dataset (Wave 7), gathered between 2017 and 2022, encompassing data from 63 countries [13]. "As per the WVS rules, every country is surveyed once per wave. All countries employed random probability representative samples of the adult population" [13]. The total sample size is 87,777.

After downloading the dataset in SPSS format, we employed binary logistic regression for data analysis. This analysis was executed using the SPSS version 28 software. Our research design is descriptive in nature. We employed binary logistic regression for each age group to ascertain the association between explanatory variables and homonegativity.

In the WVS survey, respondents were posed the following question: "Please tell me for each of the following actions whether you think it can always be justified, never be justified, or something in between." Among the actions listed as "homosexuality." Respondents were prompted to select a number between 1 and 10, with 1 representing "never justified" and 10 indicating "always justified." For the purposes of our study, we transformed this variable into a binary format. Responses ranging from 1-5 were categorized as 'Homonegative', while those between 6-10 were classified as 'Not homonegative'. Consequently, our dependent variable is labeled 'homonegative', with binary outcomes of 'Yes' and 'No'. Notably, 60,742 (69.2%) survey respondents fell into the 'homonegative' category.

Our study includes various explanatory variables: Socioeconomic Variables (income, education, employment status, and life satisfaction), Demographic Variables (age, gender, marital status, religiosity, and area of residence), and Volunteering Variables (examining the nature and extent of respondents' volunteering activities). These factors collectively shape our explanatory variables.

The only study that explored the correlation between voluntary activity and homonegativity was conducted among high schoolers [11]. It found that voluntary activities correlated with homophobia, with the activities differing for boys and girls. In our study, we intend to differentiate between men and women to see if the potential voluntary activities correlated with homonegativity differ based on gender. Since that study was limited to high schoolers, we categorized our sample into three different age groups to observe the results across different age groups. Dividing the sample into three age groups for both men and women created six age-gender categories, which are detailed (Table 1).

**Table 1:** Sample size for each gender-age group

Age	Gender	Respondents
16-29 (21,753)	Male	10,251 (11.7%)
	Female	11,502 (13.1%)
30-49 (34,839)	Male	16,192 (18.4%)
	Female	18,647 (21.2%)
50+ (31,185)	Male	15,115 (17.2%)
	Female	16,070 (18.3%)

Below, we provide a detailed account of how our socioeconomic and demographic explanatory variables were derived from the survey:

Marital status: We transformed this into a binary variable with outcomes of "Married" (or living together as if married) and "Otherwise". Religiosity: Respondents were asked whether they considered themselves religious, not religious, or atheist. Those who chose the first option were classified as ‘Religious’, while the latter two options were labeled as ‘Not Religious’. Employment: Respondents were simply asked if they had 'Paid Employment' or not. Education: Those with a university degree were categorized as having a "High Level of Education", while those with education below a university level were labeled as having a "Low Level of Education".

Income: Respondents rated their income on a scale from 1 to 10, with 1 indicating the lowest income group and 10 the highest in their country. This was transformed into a binary outcome: responses from 1 to 5 were classified as 'Low Income', while those from 6 to 10 were classified as 'High Income'. Life Satisfaction: Respondents rated their overall life satisfaction on a scale from 1 to 10. Responses from 1-5 were labeled 'Dissatisfied', and those from 6-10 as 'Satisfied'. Area of Living: This binary variable indicates whether the respondent resides in an Urban or Rural area. 

In addition to the previously introduced explanatory variables, we also included two more variables in select models:

Children: This binary variable indicates whether the respondent has any children. Freedom of political regime: Among the myriad of variables contained within the WVS data, one stands out- the "type of political regime," derived from the 2019 'Freedom House' data [14]. This variable delineates the political freedom of the respondent's country of residence, categorized into three tiers: free, partly free, and not free. For the purpose of our study, we bifurcated this into two broader categories. Countries labeled as "free" retained the designation 'Free' (comprising 37.6% of the data), while those marked as "partly free" or "not free" were grouped under 'Not Free'. The inclusion of this variable is driven by discernible variances in average homonegativity levels across different nations [15]. By integrating this variable, our goal is to ascertain the extent to which a country's political freedom predicts the homonegative sentiments of its inhabitants.

We utilized binary logistic regression for data analysis and model generation. When integrating categorical variables into logistic regression, it's essential to designate a reference category and formulate dummy variables for each of the remaining categories, setting the dummy variable to 0 for the reference category. The explanatory variables, along with their corresponding dummy variables (Table 2).

**Table 2:** Socioeconomic-demographic explanatory variables

No.	Explanatory Variable	Description	N(%) of Dummy=1
1	Marital Status: Married	Dummy set to 1 if individual is married; 0 if unmarried	55,738 (63.5%)
2	Religious Person: Religious	Dummy set to 1 if individual is religious; 0 if not religious or an atheist	53,982 (61.5%)
3	Employment Status: Paid Employment	Dummy set to 1 if individual has paid employment; 0 otherwise	52,227 (59.5%)
4	Education: University	Dummy set to 1 if individual holds a university-level degree; 0 otherwise	21,944 (25.0%)
5	Income: High Income	Dummy set to 1 if individual falls in high-income bracket; 0 for low income	32,477 (37.0%)
6	Life Satisfaction: Satisfied	Dummy set to 1 if individual is satisfied with life; 0 if dissatisfied	66,798 (76.1%)
7	Area of Living: Rural	Dummy set to 1 if individual resides in a rural area; 0 for urban area	27,474 (31.3%)
8	Children	Dummy set to 1 if individual has children; 0 if childless	61,882 (70.5%)
9	Political Regime: Free	Dummy set to 1 if individual lives in a country with a free political regime; 0 for 'not-free' political regimes	33,969 (38.7%)
Total	87,777 (100%)		

Explanatory variables regarding ‘voluntary activity’

In the WVS survey, respondents were prompted about their membership status in various voluntary organizations. The question posed was: “Now I am going to read off a list of voluntary organizations. For each organization, could you tell me whether you are an active member, an inactive member, or not a member of that type of organization?” Respondents had three choices for each of the 12 organizations: 'Not a member', 'Inactive member', and 'Active member'. We aimed to convert these options into binary variables, marking a dummy as 1 for 'Active member' and 0 for both 'Inactive member' and 'Not a member'. The rationale behind this conversion stems from the ambiguity of what 'Inactive membership' truly signifies. Does it merely denote a registered but non-participatory member or someone who occasionally engages? Due to this lack of clarity, we structured our binary variable as 'Active membership' vs. all others. These variables, along with their corresponding dummy variables (Table 3).

**Table 3:** Binary variables pertaining to voluntary membership

Variable	Description
V1: Sport or Recreational Organization	Dummy set to 1 if active member; 0 otherwise
V2: Professional Association	Dummy set to 1 if active member; 0 otherwise
V3: Art, Music, or Educational Organization	Dummy set to 1 if active member; 0 otherwise
V4: Humanitarian or Charitable Organization	Dummy set to 1 if active member; 0 otherwise
V5: Church or Religious Organization	Dummy set to 1 if active member; 0 otherwise
V6: Labor Union	Dummy set to 1 if active member; 0 otherwise
V7: Political Party	Dummy set to 1 if active member; 0 otherwise
V8: Environmental Organization	Dummy set to 1 if active member; 0 otherwise
V9: Consumer Organization	Dummy set to 1 if active member; 0 otherwise
V10: Self-help Group or Mutual Aid Group	Dummy set to 1 if active member; 0 otherwise
V11: Women’s Group	Dummy set to 1 if active member; 0 otherwise
V12: Other Organization	Dummy set to 1 if active member; 0 otherwise

Furthermore, we introduced an additional variable, termed 'V-number', that quantifies the count of voluntary organizations where the respondent is an active member. This variable is categorical, with outcomes being: 0 organizations: 50,029 (57% of respondents); 1 organization: 18,520 (21.1% of respondents); More than 1 organization: 19,228 (21.9% of respondents). The creation of 'V-number' aims to investigate if varying degrees of active volunteerism correlate with homonegativity. In this structure, the reference category is set as 0 organizations.

Data analysis

Binary logistic regression is employed when the dependent variable is binary. In our study, this binary variable is 'homonegativity.' For each age group, we formulated two models. Here, we detail the construction of Model A and Model B.

Model A

Each of the 7 primary explanatory variables(E1 to E7) and the V-number variable was individually introduced into a univariate logistic regression. The dependent variable for these regressions was 'homonegativity.' From these individual regressions, only those explanatory variables that exhibited statistical significance were shortlisted. These shortlisted variables were combined in a multivariate binary logistic regression to formulate Model A.

While constructing this model, we also evaluated the potential inclusion of variables E8 (children) and E9 (political freedom). Their incorporation was based on ensuring they did not compromise the model's fit to the data. For example, if introducing E8 led the Hosmer-Lemeshow test's significance to go above 0.05, we would omit E8, regardless of its statistical significance in the univariate logistic regression.

Model B

The primary aim of Model B is to discern the association between specific types of voluntary organizations and homonegativity. For the construction of this model, we consider only those respondents who are active members of either zero or just one organization. This decision is grounded in the rationale that for respondents with multiple memberships, pinpointing the precise organization influencing their stance on homonegativity becomes challenging. Notably, 50,029 (57%) respondents were not active in any voluntary organization, while 18,520 (21.1%) were actively engaged in just one. Model B specifically includes these subsets of respondents.

Similar to Model A, the creation of Model B commences by individually inserting the explanatory variables (including E1 to E7 and V1 to V12) into a univariate logistic regression. Variables that exhibit statistical significance are subsequently incorporated into a multivariate logistic regression. Notably, the V-number variable is omitted in Model B. Within this multivariate analysis, any explanatory variable that doesn't demonstrate statistical significance-possibly due to a confounding effect is methodically excluded. This pruning continues until all the remaining variables show statistical significance (P<0.05). While constructing this model, we also evaluated the potential inclusion of variables E8 (children) and E9 (political freedom). Their incorporation was based on ensuring they did not compromise the model's fit to the data. For example, if introducing E8 led the Hosmer-Lemeshow test's significance to go above 0.05, we would omit E8, regardless of its statistical significance in the univariate logistic regression.

Results presentation

The results section will encompass the Omnibus test, Nagelkerke R-squared value, Hosmer-Lemeshow test, and the area under the Receiver Operating Characteristic (ROC) curve for both Models A and B across each of the six gender-age groups culminating in a potential total of 12 models. Both Model A and Model B will be presented distinctly, accompanied by the relative coefficient, Wald value, and odds ratio (along with its 95% confidence interval) for all retained explanatory variables.

Handling missing data

When utilizing secondary data, the issue of missing data becomes critically important. Throughout our analysis, instances with missing data were excluded. A common threshold in research suggests that if missing data accounts for less than 20% of the dataset, it's generally considered acceptable [16].

In our Group B models, the percentage of missing data consistently fell below the 20% threshold (averaging 16.3% with a standard deviation of 2.7%). However, one exception was observed for women in age group 3, where the missing data accounted for 20.8%. In Group A Models, For the men and women in age groups 2 and 3 (comprising four models), the missing data ranged from 22% to 29%. Given some models' slightly elevated missing data percentages, we felt it prudent to conduct a sensitivity analysis.

Sensitivity analysis

For each model, we randomly excluded 25% of the data to evaluate the potential impact of missing data on our results. After running the binary logistic regression once more, we found consistent results with our initial models and equations. Moreover, these models continued to fit the data adequately.

Given our large sample size, it appears that the missing data didn't impact our models negatively. Thus, our initial concerns regarding missing data exceeding the 20% threshold in some models were assuaged.

## Results

In our study, a total of 12 models were formulated across three age groups, each analyzed separately for men and women. The results of these models are detailed in two tables. Table 4: This table displays results from the six models encompassing the categorical variable related to the 'number of active voluntary memberships'. These models are designated A + [age group] + [gender]. Table 5: This table showcases the Group B Models incorporating variables related to the 'type of voluntary organization'. Importantly, only respondents with active participation in either 0 or 1 voluntary organization were included. Models in this table are labeled as B + [age group] + [gender].

Model fitness

All formulated models demonstrated a robust fit to the data. Omnibus tests consistently exhibited statistical significance. Hosmer-Lemeshow tests remained non-significant across all models, indicating a good fit. The Area Under the Curve (AUC) of the Receiver Operating Characteristic (ROC) consistently surpassed 0.7, a threshold generally accepted as indicating satisfactory classifier performance [17]. In most models, the Nagelkerke R^2 value exceeded 24%, and no model displayed a value below 16%.

Variable representation in tables

In some models, explanatory variables E8(having children) and/or E9(free political regime) were excluded if they adversely affected model fit. Additionally, some variables were initially planned for inclusion but were later excluded due to a lack of statistical significance. In the case of exclusion, the results related to that variable are shown as hyphens (-) (Tables 4-5).

Summary of Results by Explanatory Variables (Group A Models) (Table 4)

Marital status (E1): Men: Marital status correlates with homonegativity across all age groups. Marriage decreases the odds of homonegativity by 17.8% in age group 1. However, it increases the odds by 14.7% in age group 2 and 70.1% in age group 3. Women: Only in age group 2 does marital status increase the odds of homonegativity by 31.6%.

Religiousness (E2): Religiousness is a potent predictor of homonegativity, increasing odds by 2-3 times. The association is least in men from age group 1 (1.975) and most pronounced in age group 2 (2.993).

Employment (E3): Men: No discernible association. Women: Employment reduces the odds of homonegativity by 18.4% in age group 1 and around 28% in age groups 2 and 3.

University Education (E4): Having a university education reduces the odds of homonegativity across age groups, ranging from 15.8% to 47%. The decline is most marked in age group 3, with men at 47% and women at 37.3%.

Income (E5): Having a high income decrease the odds of homonegativity across all groups, ranging from 16.9% to 42.1%. The sole exception is women in age group 1, where there's no association.

Life satisfaction (E6): Satisfaction with life reduces the odds of homonegativity across the board, with a range from 23.9% to 46.5%.

Rural area (E7): Residing in rural areas increases the odds of homonegativity across all groups, with a variance from 14.9% to 84.7%.

Children (E8): Having children boosts the odds of homonegativity in applicable age groups, ranging from 33.9% to 53.5%.

Free political regime (E9): This variable was included in 4 out of 6 models, and it indicates a strong reduction in odds of homonegativity, ranging from 70.2% to 81.3%. When present in a model, it emerges as the strongest predictor of homonegativity compared to other variables.

V-number variable: For age groups 2 and 3, an increase in the number of active memberships in voluntary organizations correlated with a decrease in homonegativity odds, especially pronounced when V > 1 (compared to V = 0).

**Table 4:** Group A models containing V-number variable The statistical significance of omnibus test for all models is <0.001. One asterisk (*) indicates that statistical significance is <0.05, while two asterisks (**) represent statistical significance being <0.01. Three asterisks (***) signify statistical significance <0.001. Hos: Hosmer–Lemeshow test significance. Neg: Nagelkerke R Square. ROC: Receiver operating characteristic. Chi-square: Chi-squared test value. B: Beta weights/regression coefficient. Wald: The Wald test value. Exp: Exponential value of B. Exp 95%CI, lower: Lower end of 95% confidence interval of exponential value of B. Exp 95%CI, upper: Upper end of 95% confidence interval of exponential value of B. Models are named as A+[age group]+ [gender]. Has uniedu: has university-level education. H-income: high income. V-number: number of active membership organizations. In some models, explanatory variables E8 (having children) and/or E9 (free political regime) were excluded if they adversely affected model fit. Additionally, some variables were initially planned for inclusion but were later excluded due to a lack of statistical significance. In the case of exclusion, the results related to that variable are shown as hyphens (-).

Age	Age group 1										Age group 2										Age group 3									
Gender	male 16-29 model A1M					Female 16-29 model A1F					Male 30-49 model A2M					Female 30-49 model A2F					Male 50 and more model A3M					Female 50 and more model A3F				
Model data	Hos 0.064	Neg 18.3%	Roc 0.727	omnibus test***		Hos 0.144	Neg 26.1%	Roc 0.766	omnibus test***		Hos 0.121	Neg 16.7%	Roc 0.717	omnibus test***		Hos 0.189	Neg 0.319	Roc 0.798	omnibus test***		Hos 0.648	Neg 16.2%	Roc 0.707	omnibus test***		Hos 0.245	Neg 28.5%	Roc 0.785	omnibus test***	
Statistical measures	B	wald	exp	Exp 95%CI:lower	Exp 95%CI:upper	B	wald	exp	Exp 95%CI:lower	Exp 95%CI:upper	B	wald	exp	Exp 95%CI:lower	Exp 95%CI:upper	B	wald	exp	Exp 95%CI:lower	Exp 95%CI:upper	B	wald	exp	Exp 95%CI:lower	Exp 95%CI:upper	B	wald	exp	Exp 95%CI:lower	Exp 95%CI:upper
married	-0.196	6.6	0.822*	0.707	0.955	-	-	-	-	-	0.137	5.8	1.147*	1.026	1.283	0.275	35.2	1.316***	1.202	1.442	0.531	121.1	1.701***	1.548	1.870	-	-	-	-	-
religious	0.681	168.3	1.975***	1.782	2.189	0.876	323.3	2.402***	2.183	2.642	1.096	659.2	2.993***	2.752	3.254	1.028	623.4	2.796***	2.579	3.031	1.002	574.1	2.723***	2.508	2.955	0.823	327.4	2.278***	2.084	2.491
paid employment	-	-	-	-	-	-0.204	17.9	0.816***	0.742	0.896	-	-	-	-	-	-0.339	55.5	0.712***	0.651	0.779	-	-	-	-	-	-0.317	49.8	0.728***	0.667	0.795
Has uniedu	-0.172	8.8	0.842**	0.751	0.943	-0.259	23.9	0.772***	0.696	0.856	-0.338	55.1	0.713***	0.652	0.780	-0.174	14.8	0.841***	0.769	0.918	-0.635	179.1	0.530***	0.483	0.581	-0.466	63.2	0.627***	0.559	0.704
H-income	-0.237	20.3	0.789***	0.711	0.874	-	-	-	-	-	-0.341	60.2	0.711***	0.653	0.775	-0.185	18.8	0.831***	0.764	0.904	-0.547	158.1	0.579***	0.532	0.630	-0.262	30.2	0.769***	0.701	0.845
Satisfied life	-0.274	18.8	0.761***	0.672	0.861	-0.276	22.8	0.759***	0.677	0.850	-0.437	69.4	0.646***	0.583	0.716	-0.405	63.2	0.667***	0.604	0.737	-0.447	67.4	0.639***	0.575	0.711	-0.454	66.8	0.635***	0.570	0.708
rural area	0.613	99.5	1.847***	1.637	2.083	0.489	80.2	1.631***	1.465	1.815	0.533	110.4	1.705***	1.543	1.883	0.331	49.0	1.392***	1.269	1.527	0.259	28.0	1.296***	1.177	1.426	0.139	7.2	1.149**	1.038	1.272
has children	0.292	11.9	1.339***	1.134	1.580	0.363	50.4	1.437***	1.300	1.589	0.429	59.8	1.535***	1.377	1.711	-	-	-	-	-	-	-	-	-	-	0.368	32.0	1.446***	1.272	1.642
Free political regime	-1.212	498.6	0.298***	0.267	0.331	-1.386	784.2	0.250***	0.227	0.276	-	-	-	-	-	-1.611	1557.4	0.200***	0.184	0.216	-	-	-	-	-	-1.675	1270.0	0.187***	0.171	0.205
V-number	-	-	-	-	-	-	-	-	-	-	-	26.4	***	-	-	-	31.3	***	-	-	-	56.9	***	-	-	-	46.3	***	-	-
V=1	-	-	-	-	-	-	-	-	-	-	-0.241	20.9	0.786***	0.708	0.871	-0.141	7.2	0.869**	0.784	0.962	-0.248	23.9	0.781***	0.707	0.862	-0.211	14.7	0.810***	0.727	0.902
V>1	-	-	-	-	-	-	-	-	-	-	-0.192	13.0	0.825***	0.744	0.916	-0.279	29.9	0.757***	0.685	0.836	-0.373	49.1	0.689***	0.621	0.765	-0.372	42.1	0.689***	0.616	0.771

Group B consists of six models tailored for individuals who are active members in either 0 or 1 voluntary organization. These models, named B+age group+gender, aim to analyze the relationship between active membership in specific types of voluntary organizations and homonegativity. The findings are detailed in (Table 5).

**Table 5:** Group B models with voluntary organization types The statistical significance of omnibus test for all models is <0.001. One asterisk (*) indicates that statistical significance is <0.05, while two asterisks (**) represent statistical significance being <0.01. Three asterisks (***) signify statistical significance <0.001. Hos: Hosmer–Lemeshow test significance. Neg: Nagelkerke R Square. ROC: Receiver operating characteristic. Chi-square: Chi-squared test value. B: Beta weights/regression coefficient. Wald: The Wald test value. Exp: Exponential value of B. Exp 95%CI, lower: Lower end of 95% confidence interval of exponential value of B. Exp 95%CI, upper: Upper end of 95% confidence interval of exponential value of B. Models are named as B+[age group]+ [gender]. Has uniedu: has university-level education. H-income: high income. V1 to V12 represent binary variables related to Voluntary Membership, as depicted in Table 3. In some models, explanatory variables E8 (having children) and/or E9 (free political regime) were excluded if they adversely affected model fit. Additionally, some variables were initially planned for inclusion but were later excluded due to a lack of statistical significance. In the case of exclusion, the results related to that variable are shown as hyphens (-).

Age	Age group 1										Age group 2										Age group 3									
Gender	male 16-29 model B1M					Female 16-29 model B1F					Male 30-49 model B2M					Female 30-49 model B2F					Male 50 and more model B3M					Female 50 and more model B3F				
Model data	Hos 0.255	Neg 21.1%	Roc 0.745	Omnibus Test ***		Hos 0.214	Neg 30.0%	Roc 0.786	Omnibus Test ***		Hos 0.890	Neg 17.4%	Roc 0.722	Omnibus Test ***		Hos 0.284	Neg 0.291	Roc 0.787	Omnibus Test ***		Hos 0.127	Neg 24.8%	Roc 0.764	Omnibus Test ***		Hos 0.211	Neg 28.8%	Roc 0.782	Omnibus Test ***	
Statistical measures	B	wald	exp	Exp 95%CI:lower	Exp 95%CI:upper	B	wald	exp	Exp 95%CI:lower	Exp 95%CI:upper	B	wald	exp	Exp 95%CI:lower	Exp 95%CI:upper	B	wald	exp	Exp 95%CI:lower	Exp 95%CI:upper	B	wald	exp	Exp 95%CI:lower	Exp 95%CI:upper	B	wald	exp	Exp 95%CI:lower	Exp 95%CI:upper
married	-0.237	5.8	0.789*	0.650	0.957	-	-	-	-	-	-	-	-	-	-	0.174	9.0	1.191**	1.063	1.334	0.333	33.9	1.395***	1.247	1.560	-	-	-	-	-
religious	0.871	168.6	2.388***	2.094	2.724	0.948	249.9	2.580***	2.294	2.902	1.055	474.1	2.873***	2.612	3.159	0.940	402.8	2.560***	2.336	2.806	0.753	214.2	2.123***	1.920	2.348	0.811	257.0	2.250***	2.037	2.484
paid employment	-	-	-	-	-	-0.259	19.0	0.772***	0.687	0.867	-	-	-	-	-	-0.362	49.8	0.696***	0.629	0.770	-	-	-	-	-	-0.331	41.9	0.718***	0.650	0.794
Has uniedu	-0.187	6.1	0.830*	0.716	0.962	-0.260	15.3	0.771***	0.677	0.878	-0.313	35.3	0.732***	0.660	0.811	-0.106	4.0	0.899*	0.811	0.998	-0.439	54.9	0.645	0.574	0.724	-0.383	29.7	0.682***	0.594	0.783
H-income	-0.179	7.1	0.836**	0.733	0.954	-	-	-	-	-	-0.302	36.4	0.740***	0.671	0.816	-0.98	4.0	0.907*	0.824	0.998	-0.416	62.2	0.660***	0.595	0.732	-0.273	25.2	0.761***	0.685	0.847
Satisfied life	-0.356	20.2	0.700***	0.600	0.818	-0.307	18.5	0.736***	0.640	0.846	-0.395	44.4	0.674***	0.600	0.757	-0.440	57.2	0.644***	0.575	0.722	-0.336	28.0	0.714***	0.631	0.809	-0.464	57.7	0.628***	0.558	0.708
rural are	0.561	50.2	1.753***	1.501	2.047	0.524	58.0	1.689***	1.476	1.933	0.506	75.2	1.658***	1.479	1.859	0.346	40.0	1.413***	1.270	1.573	0.135	5.0	1.144*	1.017	1.287	0.154	6.8	1.167**	1.039	1.310
has children	0.303	7.8	1.354**	1.094	1.675	0.291	21.0	1.337***	1.181	1.514	0.504	93.8	1.655***	1.495	1.833	0.274	18.4	1.316***	1.161	1.491	-	-	-	-	-	0.387	27.9	1.472***	1.275	1.699
Free political regime	-1.158	298.3	0.314***	0.276	0.358	-1.450	580.8	0.235***	0.209	0.264	-	-	-	-	-	-1.475	999.6	0.229***	0.209	0.251	-1.404	656.4	0.246***	0.221	0.274	-1.658	982.7	0.191***	0.172	0.211
V1: Sport	-	-	-	-	-	-0.670	20.3	0.512***	0.383	0.685	-0.738	65.4	0.478***	0.400	0.572	-0.427	10.1	0.652**	0.501	0.849	-0.665	40.1	0.514***	0.419	0.630	-0.753	25.7	0.471***	0.352	0.630
V2: Professional	-0.585	4.4	0.557*	0.323	0.962	-	-	-	-	-	-0.343	4.9	0.709*	0.523	0.963	-	-	-	-	-	-0.403	5.677	0.668*	0.479	0.931	-0.599	6.8	0.549**	0.350	0.862
V3: Art, music	-0.736	15.3	0.479***	0.331	0.693	-0.647	18.6	0.523***	0.390	0.703	-0.504	7.7	0.604**	0.424	0.862	-0.470	9.3	0.625**	0.462	0.846	-0.593	10.6	0.553*	0.387	0.789	-0.619	11.0	0.538***	0.373	0.777
V4: Humanitarian	-	-	-	-	-	-	-	-	-	-	-	-	-	-	-	-0.412	4.0	0.662*	0.443	0.990	-0.531	6.7	0.588*	0.393	0.880	-1.084	30.1	0.338***	0.230	0.498
V5: or religious org	-	-	-	-	-	-	-	-	-	-	-	-	-	-	-	-	-	-	-	-	0.193	4.2	1.213*	1.009	1.458	-	-	-	-	-
V6: Labor	-	-	-	-	-	-	-	-	-	-	-0.872	33.2	0.418***	0.311	0.563	-	-	-	-	-	-	-	-	-	-	-0.686	9.0	0.503**	0.321	0.789
V7: Political	-	-	-	-	-	-	-	-	-	-	-	-	-	-	-	-	-	-	-	-	-	-	-	-	-	-	-	-	-	-
V8: Environment	-	-	-	-	-	-	-	-	-	-	-	-	-	-	-	-	-	-	-	-	-	-	-	-	-	-	-	-	-	-
V9: Consumer	-	-	-	-	-	-	-	-	-	-	-	-	-	-	-	-	-	-	-	-	-	-	-	-	-	-	-	-	-	-
V10: Self-help	-	-	-	-	-	-	-	-	-	-	-	-	-	-	-	-	-	-	-	-	-	-	-	-	-	-	-	-	-	-
V11: Women	-	-	-	-	-	-	-	-	-	-	-	-	-	-	-	-	-	-	-	-	-	-	-	-	-	-	-	-	-	-
V12: Other	1.282	22.0	3.603***	2.109	6.155	0.531	4.1	1.701*	1.017	2.845	0.509	8.8	1.664**	1.189	2.329	0.893	17.1	2.442***	1.599	3.729	-	-	-	-	-	-	-	-	-	-

Key Associations Derived From Group B Models (Table 5)

For each age-gender group, we highlight the key associations derived from Group B Models: Men, Age Group 1: Active membership in V2 (Professional) and V3 (Art) decreases the odds of homonegativity by 41.5% and 26.4%, respectively. Conversely, V12 (Other) increases these odds by 3.6 times. Women, Age Group 1: V1 (Sport) and V3 (Art) memberships reduce the odds by 48.8% and 47.7%, respectively. V12 (Other) boosts the odds by 70.1%. Men, Age Group 2: Memberships in V1, V2, V3, and V6 (Labor Union) diminish the odds by 52.2%, 29.1%, 39.6%, and 58.2% respectively. On the flip side, V12 (Other) membership raises the odds by 66.4%. Women, Age Group 2: Participations in V1, V3, and V4 (Humanitarian) resulted in odds reductions of 34.8%, 37.5%, and 33.8%, respectively. However, V12 (Other) pushes the odds up by a factor of 2.4. Men, Age Group 3: Engaging in V1, V2, V3, and V4 decreases the odds by 48.6%, 33.2%, 44.7%, and 41.2%, respectively. In contrast, V5 (Religious Organizations) increases the odds by 21.3%. Women, Age Group 3: Involvement in V1, V2, V3, V4, and V6 reduces the odds by 52.9%, 45.1%, 46.2%, 66.2%, and 49.7% respectively. Notably, this is the only group where no voluntary activity, including V12, increases the odds of homonegativity.

## Discussion

The cautionary approach to data interpretation in our study arises from the variability in the inclusion of explanatory variables across our models. Notably, variables E8 (children) and E9 (free political regime) were integrated only when their presence didn't jeopardize the model's fit to the data. Hence, when aiming to compare variables across different models, it's essential that both models exhibit a consistent inclusion pattern, particularly concerning E8 and E9. However, it's worth noting that other variables, E1 to E7, found their way into all models as long as they demonstrated statistical significance.

To elucidate, within the framework of Group A models (Table 4), a direct comparison is viable solely between men and women in age group 1, i.e., A1M and A1F. Both these models encapsulate E8 and E9 variables while excluding the V-number variable. Transitioning to Group B models(Table 5), a similar logic applies: the data from females spanning age groups 1, 2, and 3 (i.e., B1F, B2F, B3F) and from males in age group 1 (B1M) can be juxtaposed directly, given their consistent incorporation of both E8 and E9.

When we analyze the data for men and women in age group 1, referencing Group A models (Table 4-A1M and A1F), several key insights emerge (all discussions in this paragraph pertain to age group 1). Being married or cohabiting doesn't impact homonegativity levels among women. However, there's a notable association for men, revealing a 17.8% decrease in homonegativity odds, albeit with a fairly broad 95% confidence interval (CI). In age group 1, higher income levels in men are correlated with a 21.1% reduction in homonegativity odds. Such an association isn't present among the women of this age group. Some studies have shown a negative association between income level and homonegativity [18,19]. According to the human development theory introduced by Inglehart, socioeconomic development, which often accompanies higher income levels, brings about cultural changes that promote individual autonomy, self-expression, and free choice [20]. Rising levels of existential security and autonomy fundamentally alter individuals' life experiences, causing them to prioritize goals such as freedom, which were previously less important [20]. Higher-income provides the objective resources for people to make autonomous decisions about their lives; As more importance is placed on self-expression values, individuals advocate for and defend freedom of choice in various areas of life; This includes gender roles, religious beliefs, and sexual orientation, all of which increasingly become matters of individual choice [20]. Hence, we can discern the potential influence of higher income on attitudes towards homosexuality. Women in age group 1 exhibit a more robust link between religiousness and homonegativity, with an odds ratio of 2.40, as opposed to men, who have an odds ratio of 1.97. This aligns with other studies that have identified religion as a strong predictor of homonegativity, attributable to religious doctrines that often classify homosexuality negatively [21,22]. However, it remains unclear why, in this study, this correlation is stronger in women than in men within age group 1. There's no discernible link between employment and homonegativity in men; in contrast, women in employment have an 18.4% reduced likelihood of exhibiting homonegative tendencies. Possessing a university-level education correlates with decreased homonegativity for both genders, by 15.8% for men and 22.8% for women. For both men and women in age group 1, greater life satisfaction relates to a roughly 24% reduction in homonegativity odds. Residing in rural areas is associated with increased homonegativity for both genders - a substantial 84.7% increase for men and a 63.1% increase for women.

For men, being a parent augments the odds of homonegativity by 33.9%, whereas for women, the increment is slightly higher at 43.7%. Lastly, inhabiting a nation with a free political regime emerges as a significant factor. It drastically diminishes the likelihood of homonegativity by 70.2% for men and 75% for women. Intriguingly, this variable exerts the strongest influence on homonegativity compared to all others; when you invert its odds ratio, its impact surpasses that of the religiosity variable. This may be due to the fact that free political regimes, by championing democracy, establish an environment conducive to the exchange of opinions and the cultivation of greater tolerance towards outgroups, such as homosexuals [23]. The connection between homonegativity and political freedom is rooted in self-expression values prevalent in democratic societies, which emphasize tolerance and diversity; As democracy fosters a culture of human choice and freedom, it inherently promotes tolerance towards outgroups, including homosexuals, reducing homonegativity [20]. In total, the factors influencing homonegativity discussed here derived from Group A models consistent with previous research findings [5-9].

In assessing the associations of homonegativity with specific types of voluntary organizations through Group B models(Table 5), several key relationships were identified (all discussions in this paragraph pertain to age group 1). For men in age group 1, active membership in a professional voluntary organization (V2) was linked to a 44.3% reduction in homonegativity odds, though this finding had a wide 95% confidence interval. Membership in art-music-educational organizations (V3) led to an even more significant 52.1% drop in homonegativity odds. For women in age group 1, active participation in a voluntary sports organization (V1) was associated with a 48.8% decline in the odds of homonegativity. Engaging with art-music-educational organizations (V3) mirrored this trend, with a 47.7% reduction.

Conversely, affiliation with 'other' voluntary organizations (V12) escalated homonegativity odds. Men saw this increase threefold, while women faced a 70.1% rise. Notably, the specific nature and objectives of these 'other' organizations remain unidentified. In the literature review section, we were only able to find one study that explored the association between voluntary activities and homonegativity, which was conducted on high school students [11]. Due to the age differences between the participants in that study and our current study, we can't compare the results; we can only mention them. That study [11] showed a positive correlation between homonegativity and involvement in core sports solely among boys and a negative correlation between homonegativity and non-athletic activities such as debate and science clubs solely among girls. Our study's findings concerning age group 1 reveal a negative correlation between homonegativity and active membership in sports organizations among females, as well as a negative correlation between homonegativity and active membership in art, music, and educational organizations for both genders, with odds ratios decreasing similarly (52.1% for men and 47.7% for women). Given the disparity in results between these two studies, we can speculate that age may play a role in determining which voluntary activities are associated with homonegativity among each gender. This speculation gains strength considering that our study identified different voluntary activities among the two genders, which vary across different age groups. This underscores the need for additional studies to be conducted on different age groups to obtain a clearer understanding of this matter.

When we broadened our analysis across women in age groups 1, 2, and 3 (Table 5-B1F, B2F, B3F) using Group B models, the following patterns were discernible: Membership in voluntary sports organizations diminished homonegativity odds by 48.8%, 34.8%, and 52.9% across the three age groups, respectively. Affiliation with art-music-educational organizations similarly reduced odds by 47.7%, 37.5%, and 46.2%. Professional organization membership showed a clear reduction in homonegativity only for women in the third age group, by 45.1%. Engagement with humanitarian organizations cut back homonegativity odds in age groups 2 and 3 by 37.8% and 66.2%, respectively. Active participation in a Labor Union led to a 49.7% reduction in homonegativity for women in the third age group. The enigmatic V12 ('other' voluntary organizations) augmented the odds of homonegativity by 66.4% and more than double for women in the first and second age groups, respectively. Men aged 30-50 (age group 2) saw a 58.2% reduction in homonegativity when affiliated with a Labor Union. However, no associations were spotted for men in age groups 1 and 3. Lastly, several voluntary organizations, encompassing political (V7), environmental (V8), consumer (V9), self-help (V10), and women-oriented ones (V11), showed no discernible relationship with homonegativity in any of the models under scrutiny.

Expanding on the findings from Group A models (Table 4), individuals in age groups 2 and 3 who maintain active membership in voluntary organizations exhibit a decrease in homonegativity odds ranging from 14-22%. Interestingly, holding memberships across multiple organizations amplifies this effect: those in age groups 2 and 3 show an 18-31% reduction in homonegativity odds, indicating that diverse engagements might foster more inclusive mindsets.

While these results undeniably highlight the negative correlation between active membership in voluntary organizations and homonegativity, it's crucial to distinguish carefully between correlation and causation. The data doesn't definitively argue that voluntary participation directly mitigates homonegativity. However, considering the evidence, it remains a viable theory. Supporting this potential causative link, the literature shows that volunteering cultivates a richer environment for interactions, stimulating dialogues that create safe havens for self-expression. Such environments foster mutual understanding and tolerance [24].

Moreover, active participation in voluntary organizations has been linked to developing generalized trust, mutual respect, and tolerance [25]. Given these premises, it stands to reason that if voluntary engagements bolster general tolerance, they might very well also influence a more accepting stance towards homosexuality. Active membership in voluntary organizations increases the chances of individuals meeting new people, including those from the LGBTQ+ community. This interaction can further lower homonegativity [26]. This aligns with Allport's contact hypothesis, which posits that increased interaction with diverse groups can mitigate prejudice [27].

Additionally, the culture within these organizations often promotes inclusive values, which can lead to an alignment of individual attitudes with those progressive norms, further diminishing homonegative sentiments [28]. Within the framework of Robert Putnam's theory in "Bowling Alone," active membership in voluntary organizations plays a crucial role in revitalizing social capital, which can foster greater social equality and reduce prejudice [29]. This engagement in community activities suggests a pathway to diminishing homonegativity, as stronger social bonds and increased civic participation contribute to a more inclusive and accepting society. Consequently, it seems plausible that active involvement in such organizations encourages greater acceptance of minorities, including the LGBTQ+ community. This underlying correlation could indeed hint at a causative relationship, making the findings of this study all the more significant.

Our study possesses strengths as well as limitations. The strengths include: 1) Utilization of a large and diverse dataset: The study's utilization of a substantial dataset from multiple countries enhances the generalizability and robustness of its findings, setting it apart from many other studies on homonegativity; 2) Use of binary logistic regression: Employing binary logistic regression allowed for a comprehensive analysis of the relationships between variables and homonegativity, providing a detailed understanding of influencing factors; 3) Valuable insights into factors influencing homonegativity: The study offers valuable insights into the factors associated with homonegativity, shedding light on the association between active participation in voluntary organizations and homonegative attitudes.

Our study's limitations include: 1) reliance on secondary data: This led to a lack of control over data quality. Missing data exceeded the 20% threshold in certain age groups for Group A models but did not exceed 29%. Additionally, the V12 variable lacked specificity regarding types of organizations. The inconsistent inclusion of variables E8 and E9 hindered comprehensive model comparisons, as they were excluded from some models due to their negative impact on model fit (explained in detail in the data analysis section). 2. Generalizability of results: Although our study benefits from a large dataset and diverse sample size, it's important to recognize potential limitations in applying our findings to specific populations or contexts.

Future research directions: Recommendations for future studies could include exploring different methodologies and focusing on specific demographic groups to address limitations of the current study and deepen understanding of factors related to homonegativity, particularly active voluntary participation.

## Conclusions

Demographic and socioeconomic variables play a discernible role in shaping attitudes toward homonegativity. However, their impact varies across distinct gender-age groups. Notably, higher life satisfaction tends to diminish homonegative sentiments. Furthermore, the political freedoms characterizing a respondent's home country emerge as the most potent predictor, inversely related to homonegativity.

Our analysis has further illuminated the relationship between active participation in various voluntary organizations and homonegativity. Specifically, engagements with sports or recreational organizations, professional associations, art, music, or educational organizations, as well as humanitarian or charitable entities often correlate with reduced homonegative tendencies among certain gender-age groups. Notably, every gender-age group highlighted in the study had at least two voluntary organizations where active participation was linked to decreased homonegativity.

## References

[1] Rye BJ, Meaney GJ (2010). Measuring homonegativity: A psychometric analysis. Can J Behav Sci.

[2] Thepsourinthone J, Dune T, Liamputtong P, Arora A (2021). Out of the closet, not yet out of the house: Gay men’s experiences of homonegativity and internalized homonegativity. Healthcare (Basel).

[3] Plöderl M, Tremblay P (2015). Mental health of sexual minorities. A systematic review. Int Rev Psychiatry.

[4] Michli S, Jamil FE (2022). Internalized homonegativity and the challenges of having same-sex desires in the Lebanese context: A study examining risk and protective factors. J Homosex.

[5] Meladze P, Brown J (2015). Religion, sexuality, and internalized homonegativity: Confronting cognitive dissonance in the Abrahamic religions. J Relig Health.

[6] Doebler S (2015). Relationships between religion and two forms of homonegativity in Europe-A multilevel analysis of effects of believing, belonging and religious practice. PLoS One.

[7] Watt L, Elliot M (2019). Homonegativity in Britain: Changing attitudes towards same-sex relationships. J Sex Res.

[8] Stulhofer A, Rimac I (2009). Determinants of homonegativity in Europe. J Sex Res.

[9] Marsh T, Brown J (2011). Homonegativity and its relationship to religiosity, nationalism and attachment style. J Relig Health.

[10] Güntert ST, Wehner T, Mieg HA (2022). Definition of volunteer work and a model of volunteer activity. Organizational, Motivational, and Cultural Contexts of Volunteering.

[11] Osborne D, Wagner III WE (2007). Exploring the relationship between homophobia and participation in core sports among high school students. Sociol Perspect.

[12] (2023). WVS database. https://www.worldvaluessurvey.org/WVSContents.jsp.

[13] Haerpfer Haerpfer, C. C., Inglehart Inglehart (2023). World values survey wave 7 (2017-2022) cross-national data-set. World Values Survey Association.

[14] (2023). Countries and territories. https://freedomhouse.org/countries/freedom-world/scores.

[15] Jäckle S, Wenzelburger G (2015). Religion, religiosity, and the attitudes toward homosexuality—A multilevel analysis of 79 countries. J Homosex.

[16] Sutthipong M (2016). Logistic regression with missing data: A comparison of handling methods, and effects of percent missing values. JTLE.

[17] Horng-Shing Lu H, Schölkopf B, Zhao H (2011). ROC graphs: Notes and practical considerations for data mining researchers. Handbook of Statistical Bioinformatics.

[18] Zhou M, Hu T (2019). Social tolerance of homosexuality: A quantitative comparison of mainland China, Singapore, and Taiwan. Chin Sociol Rev.

[19] Slenders S, Sieben I, Verbakel E (2014). Tolerance towards homosexuality in Europe: Population composition, economic affluence, religiosity, same-sex union legislation and HIV rates as explanations for country differences. Int Sociol.

[20] Inglehart R, Welzel C (2005). Modernization, Cultural Change, and Democracy The Human Development Sequence. Cambridge University Press.

[21] Putnam RD (2000). Bowling Alone: The Collapse and Revival of American Community. https://books.google.co.th/books/about/Bowling_Alone.html?id=rd2ibodep7UC&redir_esc=y.

[22] Wolf JK, Platt LF (2022). Religion and sexual identities. Curr Opin Psychol.

[23] Yip AK (2005). Queering religious texts: An exploration of British non-heterosexualChristians’and Muslims’strategy of constructing sexuality-affirming hermeneutics. Sociology.

[24] Hu F, Lee IC (2018). Democratic systems increase outgroup tolerance through opinion sharing and voting: An international perspective. Front Psychol.

[25] Rusu DE (2016). Volunteering and social interactions towards self-development. The 4th International Virtual Conference on Advanced Scientific Results.

[26] Wilson J, Musick M (1999). The effects of volunteering on the volunteer. Law Contemporary Problems.

[27] Herek GM (2007). Confronting sexual stigma and prejudice: Theory and practice. Social Issues.

[28] Allport GW (1954). The Nature of Prejudice. https://psycnet.apa.org/record/1954-07324-000.

[29] Schein EH (2010). Organizational Culture and Leadership. https://books.google.co.th/books/about/Organizational_Culture_and_Leadership.html?id=DlGhlT34jCUC&redir_esc=y.

